# Green Biosynthesis of Silver Nanoparticles Using Leaf Extract of *Carissa carandas* L. and Their Antioxidant and Antimicrobial Activity against Human Pathogenic Bacteria

**DOI:** 10.3390/biom11020299

**Published:** 2021-02-17

**Authors:** Reetika Singh, Christophe Hano, Gopal Nath, Bechan Sharma

**Affiliations:** 1 Department of Biochemistry, University of Allahabad, Allahabad 211002, India; 2 Laboratoire de Biologie des Ligneux et des Grandes Cultures (LBLGC), INRA USC1328, Université d’Orléans, Eure et Loir Campus, 21 rue de Loigny la Bataille, F-28000 Chartres, France; 3 Bioactifs et Cosmétiques, Centre National de la Recherche Scientifique (CNRS)—Groupement de Recherche 3711, Université d’Orléans, 45067 Orléans CEDEX 2, France; 4 Department of Microbiology, Institute of Medical Sciences, Banaras Hindu University, Varanasi 221005, India; gopalnath@gmail.com

**Keywords:** green biosynthesis, nanoparticles, antioxidant, antibacterial, *Carissa carandas* L.

## Abstract

*Carissa carandas* L. is traditionally used as antibacterial medicine and accumulates many antioxidant phytochemicals. Here, we expand this traditional usage with the green biosynthesis of silver nanoparticles (AgNPs) achieved using a *Carissa carandas* L. leaf extract as a reducing and capping agent. The green synthesis of AgNPs reaction was carried out using 1mM silver nitrate and leaf extract. The effect of temperature on the synthesis of AgNPs was examined using room temperature (25 °C) and 60 °C. The silver nanoparticles were formed in one hour by stirring at room temperature. In this case, a yellowish brown colour was developed. The successful formation of silver nanoparticles was confirmed by UV–Vis, Fourier transform infrared (FT-IR) and X-ray diffraction (XRD) analysis. The characteristic peaks of the UV-vis spectrum and XRD confirmed the synthesis of AgNPs. The biosynthesised AgNPs showed potential antioxidant activity through DPPH assay. These AgNPs also exhibited potential antibacterial activity against human pathogenic bacteria. The results were compared with the antioxidant and antibacterial activities of the plant extract, and clearly suggest that the green biosynthesized AgNPs can constitute an effective antioxidant and antibacterial agent.

## 1. Introduction

Nanotechnology is emerging and exponentially growing field with wide applications in science and technology [1,2,3,4,5,6,7]. Nanotechnology is an interdisciplinary area of research using core techniques of various disciplines like chemistry, engineering, physics and biological sciences, and leading to the development of novel strategies to manipulate minute particles resulting in the production of nanoparticles (NPs). These NPs may be defined as particles with at least one dimension ranging from 1–100 nm. Nanotechnology deals with the synthesis, development and applications of a variety of NPs. Generally, nanoparticles are prepared using noble metals such as gold, silver, platinum etc., through a variety of chemical and physical methods [8,9], but these methods are not eco-friendly [10,11,12]. There is an urgency to develop an eco-friendly process for the nanoparticle synthesis that does not employ toxic chemicals. Nowadays, green biosynthesis methods are commonly used for the development of NPs using various biological systems such as yeast, fungi, bacteria and plant extracts [13,14,15]. Among them, plant extract-based biosynthesis of silver nanoparticles with controlled physicochemical properties has been reported by many researchers [16,17]. Recent reports included the biosynthesis of silver nanoparticles using *Ocimum basilicum* [10], *Silybum marianum* [11], *Azadirachta indica* [18], *Boerhaavia diffusa* [19], *Bergenia ciliate* [20], *Elephantopus scaber* [21] and *Urtica dioica* [22]. Silver nanoparticles prepared using biological materials have the properties of high surface area, small size and high dispersion. Since olden times, silver has been well known for its antibacterial activity for many bacterial strains [23,24]. The high antibacterial potential of silver nanoparticles is a result of high surface expansion providing maximum contact with the environment [25]. For the various applications of AgNPs, controlled development regarding size and shape, as well as the stability of the silver nanoparticles, is important [26].

*Carissa carandas* is commonly known as crane berry and belongs to the family Apocynaceae. *C. carandas* contain a wide range of active phytocompounds, and research is still finding uses and applications for them as they are rich in flavonoids, vitamin C and iron. In particular, leaf extracts of this plant are well-known and traditionally used for their high antioxidant and antimicrobial potential [27,28]. It is well known that flavonoids are natural and potential antioxidants [29].

In the present study, a biosynthesis method was adopted due to its eco-friendly nature. A novel approach was made using *Carissa carandas* L. leaves for AgNPs synthesis. This is the first report to use the green chemistry route for the synthesis of AgNPs from *C. carandas* leaves and to explore their antioxidant and antibacterial activity.

## 2. Materials and Methods

### 2.1. Plant Materials and Chemicals

The *C. carandas* leaves were collected from wild grown plants located at Fatehpur, UP, India. All the chemicals used were of analytical grade and purchased from Sigma-Aldrich (Saint Quentin Fallavier, France). Millipore distilled water was used for the experiments. All the experiments were done in triplicate.

### 2.2. Extract Preparation

Fresh healthy leaves were collected from a wild grown plant. Leaves were washed properly under running tap water to remove dust particles and also rinsed with Millipore distilled water. Leaves (4 g) were finely cut and boiled for 2 min. in 40 mL double distilled water (DDW). The solution was filtered using Whatmann no. 1 filter paper and used for nanoparticle synthesis and antimicrobial assay. Extracts were completely protected from light exposure during biosynthesis and after.

### 2.3. Synthesis of Silver Nanoparticles

A freshly prepared aqueous solution of silver nitrate (AgNO_3_, 1 mM) was used for AgNPs synthesis. Plant extracts (5 mL) were mixed with 45 mL of silver nitrate solution. To observe the effect of temperature on the synthesis of silver nanoparticles, biosynthesis was carried out at room temperature (25 °C) and 60 °C.

### 2.4. Characterization of Silver Nanoparticles

#### 2.4.1. UV-Vis Spectroscopic Analysis

UV-Vis spectroscopic analysis of biosynthesised AgNPs was performed by continuous scanning from 350 to 700nm (Thermo-Scientific – Evolution- 201, Illkirch, France) and 1 mM silver nitrate solution was used for baseline correction.

#### 2.4.2. X-ray Diffraction (XRD) Analysis

X-ray diffraction (XRD) analysis of purified AgNPs was performed using an XRD-6000 X-ray diffractometer (Shimadzu, Kyoto, Japan). The XRD machine was operated at a voltage of 40 kV and a current of 30 mA with Cu Kα radiation in θ–2θ configurations. The average size of AgNPs was calculated using the Debye-Scherrer equation by determining the width of the (1 1 1) Bragg reflection [30].

#### 2.4.3. Fourier Transform Infrared (FT-IR) Analysis

Characterisation of nanoparticles was done using an FTIR (Bruker- Alpha, V70, Palaiseau, France) spectrometer. The fine powder of nanoparticles was analyzed using FTIR (in the range of 500–4000 cm^−1^) to study the biomolecules presence as capping agents on silver NPs surface.

### 2.5. Free Radical Scavenging Activity

Free radical scavenging activity was calculated using the method used by Bhakya et al. with slight modifications [31,32]. Samples (1 mL) contains different concentrations (50, 100, 150, 200, 250, 300, 350 and 400 µL/mL) of AgNPs, were mixed with 1 mL of freshly prepared DPPH (0.004% (*w*/*v*) in absolute methanol) solution and mixed properly. The reaction solution was incubated for 30 min in the dark at room temperature. The absorbance was recorded at 517 nm using a UV-vis spectrophotometer (Multiskan GO, Thermo Fischer Scientific, Illkirch, France). Methanol was used as a blank and DPPH was used as a control. Free radical scavenging activity was expressed as the percentage of inhibition, which was determined using the following formula [33]:

$$\%\;\mathrm{of}\;\mathrm{free}\;\mathrm{radical}\;\mathrm{scavenging}\;\mathrm{activity}\;=\;\frac{Ac-As}{Ac}\;\times \;100$$

where *Ac* is the absorbance of control and *As* is the absorption of experimental sample.

### 2.6. Analysis of Antibacterial Sensitivity Test

The antibacterial activities of the extract, silver nitrate and AgNPs were determined against *Salmonella typhimurium, Enterobacter faecalis, Shigella flexneri, Citrobacter spp.* and *Gonococci spp.* using a disc diffusion method [34]. Pure young cultures of bacteria were obtained by subculturing the bacteria on agar-solidified Luria broth (LB) medium. The bacterial suspension (0.5 McFarland’s) was swabbed onto the agar plates using a swab stick. 5 µL of extract, AgNPs and AgNO_3_ were dropped onto sterile discs (5 mm) with AgNO_3_ was used as a control. The cultured plates were incubated for overnight in the incubator (Heratherm Compact Thermo Scientific, Illkirch, France) at 37 °C. An ordinary scale was used to measure the inhibition zone (mm) around the disc.

### 2.7. Determination of Minimum Inhibitory Concentration (MIC)

The MIC was calculated in a similar way as earlier reported [32,33,34,35,36]. The MIC of AgNPs (synthesized at 25 °C) was determined using a broth microdilution method. Bacterial suspension (20 µL) was poured into each well of 96 well U bottom microtiter plates, then different concentrations (20–180 µL) of AgNPs and AgNO_3_ were used for MIC experiments. The microtiter plate was incubated at 37 °C for 24 h.

### 2.8. Statistical Analysis

The experiments were performed in triplicate, and for statistical analysis XL-stat_2018 (Addinsoft, Paris, France) was used.

## 3. Results

### 3.1. Formation of Silver Nanoparticles

**Figure 1:**
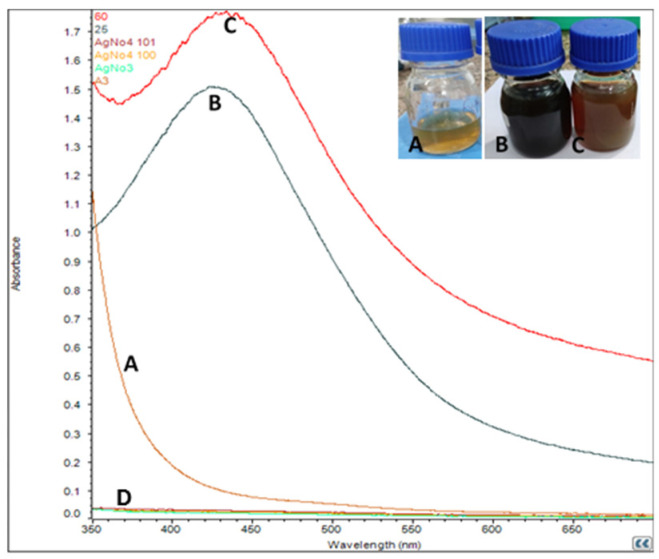
UV-vis absorption spectrum of (A) AgNO_3_, (B) biosynthesized silver nanoparticles at 25 °C and (C) at 60 °C, and *Carissa carandas* leaf extracts (D, green line).

Formation of AgNPs started just after mixing the leaf extract into the AgNO_3_ solution (1 mM). After the addition of extracts, the AgNO_3_ started to change colour from colourless to yellowish brown in about 15–20 min, and finally the solution turned to dark brown colour in about 60–65 min at room temperature. At high temperature (60 °C), a yellowish colour appeared in 10–15 min and a dark brown colour appeared within 50 min (Figure 1). The pH values of AgNPs at 25 °C and 60 °C were 7.6 and 7.2, respectively.

### 3.2. UV-vis Spectroscopy Analysis

Formation of AgNPs was again confirmed using UV–visible spectral analysis. The characteristic surface plasmon resonance (SPR) absorption band of biosynthesized AgNPs was obtained at 432 nm and 444 nm for reactions carried out at 25 °C and 60 °C, respectively (Figure 1).

### 3.3. XRD Analysis

**Figure 2:**
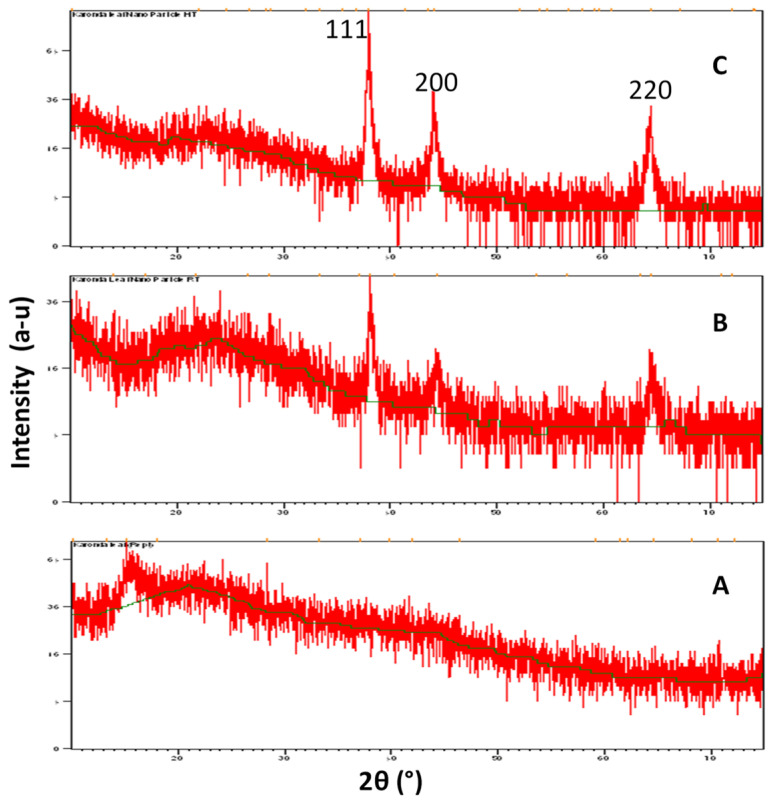
X-Ray Diffraction spectrum of (**A**) *Carissa carandas* leaf extract, (**B**) biosynthesized silver at 25 °C and (**C**) at 60 °C.

XRD analysis of the nanoparticles showed strong peaks corresponding to (1 1 1), (2 0 0) and (2 2 0) Bragg reflection based on the face-centred cubic structure of AgNPs (Figure 2). These planes confirmed the crystalline nature of the AgNPs. These peaks (111, 200 and 220) represented the 2θ° values 38.06, 44.23 and 67.43, respectively. The average crystallite size, as calculated by Scherer’s equation, was found to be 35 ± 2 nm (25 °C) and 30 ± 3 nm (60 °C).

### 3.4. FT-IR Analysis

**Figure 3:**
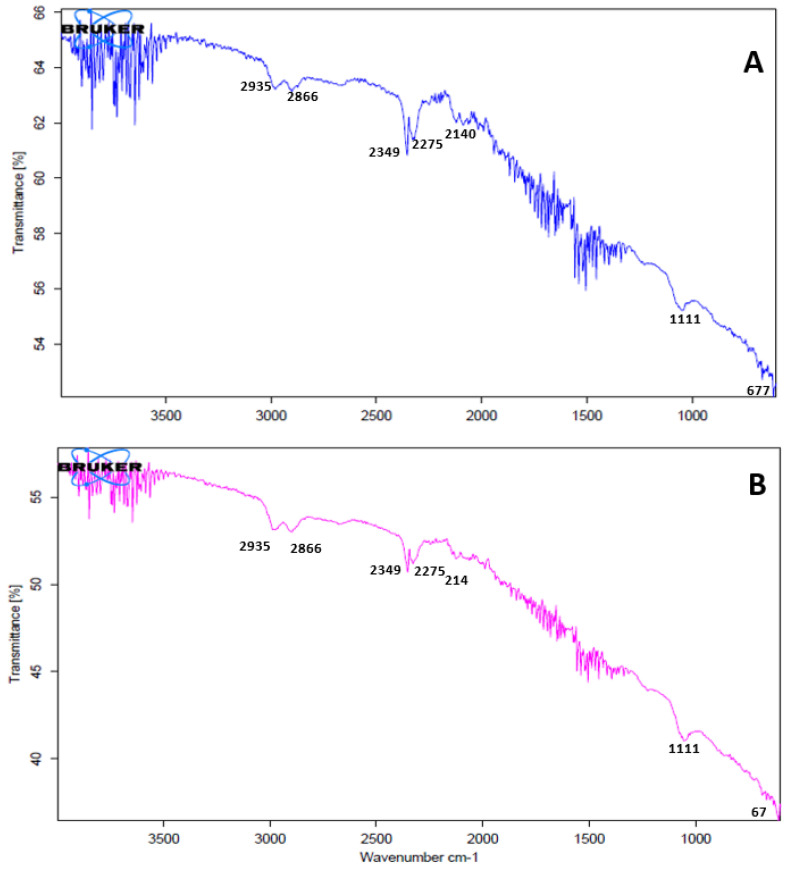
Fourier transform infrared (FT-IR) spectrum of *C. carandas* leaf extract-mediated biosynthesized silver at 25 °C (**A**) and at 60 °C (**B**).

FT-IR measurements were carried out to identify the possible biomolecules responsible for the reduction of the Ag^+^ ions and capping of the bioreduced silver nanoparticles synthesized by *C. carandas* leaf extracts. Figure 3 represents the FT-IR spectrum of nanoparticles synthesized at 25 °C and 60 °C. FT-IR peaks showed the different functional groups. Silver nanoparticles synthesized at 25 °C and 60 °C showed almost similar absorption peaks in regions already related to the presence of polyphenols capped by AgNPs [11]. The FT-IR spectra had major vibration modes at 677, 1111, 2140, 2275, 2349, 2866 and 2935. All these spectra represented different functional groups. FT-IR studies suggested the presence of various groups of secondary metabolites.

### 3.5. Antioxidant Activity

The result showed the antioxidant potential of AgNPs. Antioxidant activity of AgNPs increased from 5-15% of concentrations. Maximum DPPH free radical scavenging antioxidant activity was found at a 15% NPs concentration, reaching antioxidant capacity of 90.3% and 87.12% for AgNPs synthesized at 25 °C and 60 °C, respectively (Table 1). After 15% of sample concentration, antioxidant activity started decreasing with higher concentrations. For comparison, the DPPH antioxidant activity obtained for *C. carandas* leaf extract (5%) and silver nitrate (5%) were 12.65 ± 0.30% and 34.81 ± 0.50%, respectively, whereas the DPPH antioxidant activity of the synthetic antioxidant butylated hydroxyanisole (BHA, 50 µM) was 68.12 ± 1.27%.

### 3.6. Antibacterial Sensitivity Test

**Figure 4:**
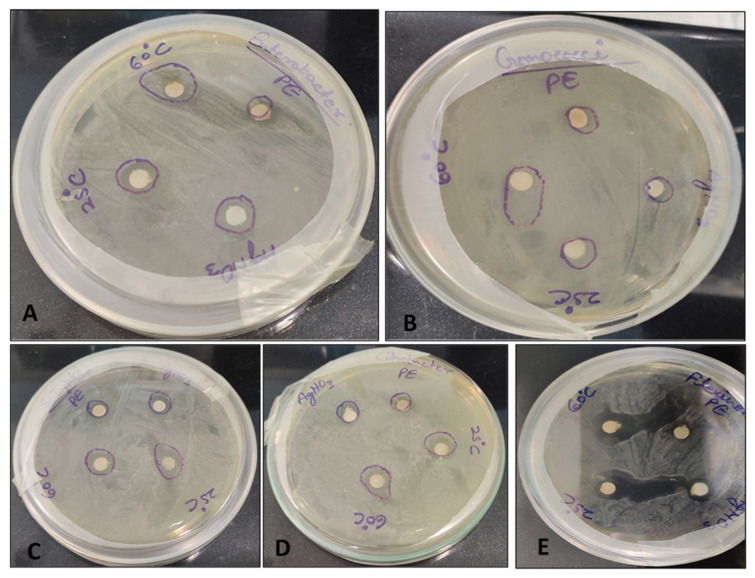
Zone of inhibition of silver nanoparticles against human pathogenic bacteria. (**A**) *Enterobacter faecalis,* (**B**) *Gonococci spp.,* (**C**) *Salmonella Typhi,* (**D**) *Citrobacter spp.,* (**E**) *Shigella flexineri*.

AgNPs showed the highest bacterial growth inhibition compared with the plant extract (PE) and AgNO_3_ (Figure 4). Maximum growth inhibition was observed against *Shigella flexineri* (24 mm).

The result showed the antimicrobial activity of PE, AgNO_3_ and AgNPs (Table 2).

### 3.7. Determination of Minimum Inhibitory Concentration (MIC)

**Figure 5:**
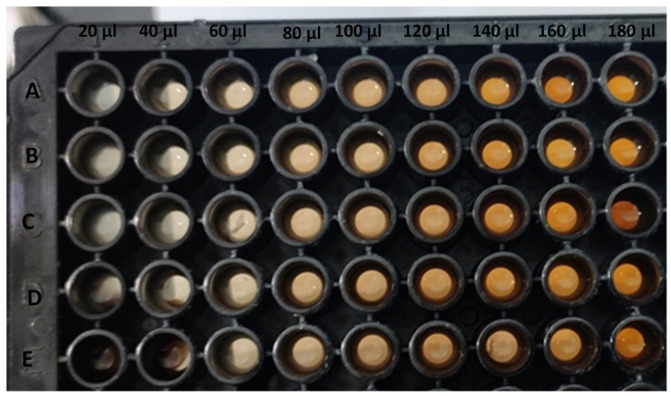
Image representing the minimum inhibitory concentration of AgNPs. (**A**) *Enterobacter faecalis,* (**B**) *Gonococci spp.,* (**C**) *Salmonella typhimurium,* (**D**) *Shigella flexneri,* (**E**) *Citrobacter spp.*

The results showed the MIC values of AgNPs against selected bacteria varied, and the MIC value depended upon the bacterial strains (Figure 5).

MIC value for *Shigella flexneri* was 60 µL and for *Citrobacter spp.* (80 µL), while for other bacteria the MIC value was observed with 100 µL.

## 4. Discussion

Colour changes of AgNO_3_ solutions confirmed the synthesis of nanoparticles [11,37,38]. The AgNO_3_ solutions changed their color from colourless to yellowish brown and finally to dark brown. Synthesis of AgNPs at room temperature took more time when compared to synthesis at 60 °C [37]. This may be due to the high temperature. Previous reports explained that colour changes of the solution showed the presence of AgNPs due to excitation of surface plasmon vibrations [38]. Rapid consumption of the reactants ultimately develops the formation of smaller nanoparticles [39,40]. UV-vis spectrum analysis also confirmed the synthesis of AgNPs. Plant extracts act as reductants for AgNO_3_, and finally the formation of AgNPs takes place. Metal nanoparticles, such as silver, have free electrons, which develop the SPR absorption band [41]. AgNPs formed at 60 °C showed a higher absorbance peak, indicating a higher number of nanoparticles synthesized at 60 °C. Similar observations have been reported by the several researchers [10,11,17,42]. Formation of nanoparticles was confirmed by the broadening of Bragg’s peaks. The XRD patterns obtained were similar to those in earlier reports [10,11,17,37]. The XRD peaks were very intense and showed that the AgNPs were synthesized under a nanoregime, having a crystalline nature. A similar pattern of peaks was also reported by a number of researchers [43,44]. The FT-IR spectrum showed the presence of different functional groups and also revealed the presence of various phytochemicals in the plants. Vibration bands showed similar pattern to those reported by Joshi et al. 2018 [45]. The FT-IR spectrum band at 677, 1111 represents the vibration of C=C alkenes and the presence of the methoxy group (-OCH_3_). Vibration bands 2935 and 2866 showed the presence of -C-H- stretching of aromatic rings and the C-O group, respectively. Spectrum peaks 2140, 2275 and 2349 proved the presence of alkynes N=C=O and O=C=O, respectively. Most of the vibration bands were similar to those in previous reports [45]. This study proved the presence of various phytochemical groups in Karonda leaf extracts.

*C. Carandas* is a medicinal plant traditionally used for its antioxidant and antimicrobial activities in various traditional medicines [27,28]. Here, by using it for green biosynthesis of antioxidant and antimicrobial AgNPs, we decided to revisit these traditional uses of the *C. carandas* leaf extract. The obtained AgNPs showed high antioxidant potential. The DPPH radical is typically used to show the presence of antioxidants in sample/materials [11]. The *C. carandas* leaf extract is described as having a high accumulation of flavonoids [27,28]. When using a plant extract during green biosynthesis of AgNPs, flavonoids can cap the AgNPs and thus increase their antioxidant activity [10,11]. Higher antioxidant activity of AgNPs was also reported in many plant-based synthesized AgNPs [7,12]. In agreement with our observation, the antioxidant power of AgNPs is known to vary with the concentration applied [44]. Results showed high antibacterial activity of AgNPs against all the selected bacteria. AgNPs showed the highest inhibition zone in comparison to the plant extract and AgNO_3_. The antibacterial activity showed that AgNPs biosynthesized using a *C. carandas* leaf extract were more likely to inhibit the growth of gram-negative bacteria, especially against *Shigella flexineri*. Silver is well known for its antibacterial activity [10]. Antibacterial activity of plant-based AgNPs has been reported by many workers [10,11,16,17,21,46,47]. It has been proposed that antibacterial activity can be caused by perforation of the microbial cell membrane [10].

## 5. Conclusions

A *Carissa carandas* leaf extract was successfully used for the biosynthesis of silver nanoparticles. Formation of silver nanoparticles was confirmed through UV-vis spectrum analysis, XRD and FT-IR studies. Biosynthesized silver nanoparticles showed potential free radical scavenging activity and bacterial growth inhibition. The antibacterial activity showed that AgNPs biosynthesized using a *C. carandas* leaf extract were more likely to inhibit growth gram-negative bacteria, especially against *Shigella flexineri*. Antioxidant potential of these nanoparticles can be used for many free radicals scavenging applications. For this purpose, there is need of extensive research, especially in in vivo conditions, to find out the precise dose and toxicity evaluation before the application of these nanoparticles. Future research will be conducted to identify end-capping phytochemicals responsible for these biological activities.

## Figures and Tables

**Table 1:** Antioxidant activity.

Concentration (%NPs/mL)	AgNPs (25 °C)	AgNPs (60 °C)
5	86.45 ± 0.38 cd	76.69 ± 0.68 i
10	88.89 ± 0.64 b	85.54 ± 0.76 de
15	90.30 ± 0.35 a	87.12 ± 045 c
20	82.67 ± 0.34 f	84.62 ± 0.68 e
25	80.35 ± 0.44 g	82.12 ± 0.73 f
30	76.02 ± 0.38 i	78.58 ± 0.83 h
35	72.84 ± 1.00 j	76.26 ± 0.91 i
40	70.53 ± 0.49 k	74.25 ± 0.90 j

Values are means and standard deviations of three independent data sets. Different letters represent significant differences between the various extraction conditions (*p* < 0.05). Values are means and standard deviations of three independent data sets. Different letters represent significant differences between the various extraction conditions (*p* < 0.05) for each bacterial strain.

**Table 2:** Bacterial growth inhibition potential of biosynthesized AgNO_3_ from *C. carandas* leaf extract.

Bacteria	Growth Inhibition Zone (mm)			
	Plant Extract	AgNO_3_	AgNPs (25 °C)	AgNPs (60 °C)
*Enterobacter faecalis*	7.0 ± 0.0 d	9.0 ± 0.6 c	10.0 ± 0.0 b	16.0 ± 1.0 a
*Gonococci spp.*	6.0 ± 0.0 d	8.0 ± 0.00 c	9.0 ± 0.0 b	14.3 ± 0.6 a
*Salmonella typhimurium*	8.0 ± 1.0 c	10.0 ± 1.0 bc	14.0 ± 0.0 a	12.0 ± 1.0 b
*Citrobacter spp.*	8.0 ± 1.0 c	10.0 ± 0.0 b	12.0 ± 1.0 a	14.0 ± 1.0 a
*Shigella flexneri*	8.0 ± 1.0 d	11.0 ± 1.0 c	24.0 ± 1.0 a	21.3 ± 0.6 b

Values are means and standard deviations of three independent data sets. Different letters represent significant differences between the various extraction conditions. (*p* < 0.05) for each bacterial strain. Ciprofloxacin was used as a standard antibiotic, showing an inhibition zone of 26.55 ± 0.25 mm.

## Data Availability

The data presented in this study are available on request from the corresponding author.
